# R-Syst::diatom: an open-access and curated barcode database for diatoms and freshwater monitoring

**DOI:** 10.1093/database/baw016

**Published:** 2016-03-17

**Authors:** Frédéric Rimet, Philippe Chaumeil, François Keck, Lenaïg Kermarrec, Valentin Vasselon, Maria Kahlert, Alain Franc, Agnès Bouchez

**Affiliations:** 1 INRA—UMR Carrtel, 75 Av. De Corzent—BP 511, Thonon Les Bains Cedex FR-74203, France; 2 UMR CARRTEL, University of Savoie, Le Bourget Du Lac FR-73370, France; 3 INRA, UMR BioGeCo, 69 Route D’arcachon, Cestas Cedex FR-33612, France; 4 University of Bordeaux 1, UMR BioGeCo, Talence FR-33400, France; 5 ASCONIT Consultants, Naturopôle—Bât. C, 3, Bd De Clairfont, Toulouges FR-66350, France, and; 6 Department of Aquatic Sciences and Assessment, Swedish University of Agricultural Sciences, PO Box 7050, Uppsala SE-750 07, Sweden

## Abstract

Diatoms are micro-algal indicators of freshwater pollution. Current standardized methodologies are based on microscopic determinations, which is time consuming and prone to identification uncertainties. The use of DNA-barcoding has been proposed as a way to avoid these flaws. Combining barcoding with next-generation sequencing enables collection of a large quantity of barcodes from natural samples. These barcodes are identified as certain diatom taxa by comparing the sequences to a reference barcoding library using algorithms. Proof of concept was recently demonstrated for synthetic and natural communities and underlined the importance of the quality of this reference library. We present an open-access and curated reference barcoding database for diatoms, called R-Syst::diatom, developed in the framework of R-Syst, the network of systematic supported by INRA (French National Institute for Agricultural Research), see http://www.rsyst.inra.fr/en. R-Syst::diatom links DNA-barcodes to their taxonomical identifications, and is dedicated to identify barcodes from natural samples. The data come from two sources, a culture collection of freshwater algae maintained in INRA in which new strains are regularly deposited and barcoded and from the NCBI (National Center for Biotechnology Information) nucleotide database. Two kinds of barcodes were chosen to support the database: 18S (18S ribosomal RNA) and *rbc*L (Ribulose-1,5-bisphosphate carboxylase/oxygenase), because of their efficiency. Data are curated using innovative (Declic) and classical bioinformatic tools (Blast, classical phylogenies) and up-to-date taxonomy (Catalogues and peer reviewed papers). Every 6 months R-Syst::diatom is updated. The database is available through the R-Syst microalgae website (http://www.rsyst.inra.fr/) and a platform dedicated to next-generation sequencing data analysis, virtual_BiodiversityL@b (https://galaxy-pgtp.pierroton.inra.fr/). We present here the content of the library regarding the number of barcodes and diatom taxa. In addition to these information, morphological features (e.g. biovolumes, chloroplasts…), life-forms (mobility, colony-type) or ecological features (taxa preferenda to pollution) are indicated in R-Syst::diatom.

Database URL: http://www.rsyst.inra.fr/

## Introduction

Microalgae are the dominant primary producers of aquatic ecosystems. They display a huge taxonomic diversity—the numbers of diatoms alone have been estimates at 100 000 species (1) and each taxon occupies a particular ecological niche (2). These properties make them excellent ecological indicators. One of the most often used algal class for ecological assessment is diatoms (3). The first studies demonstrating the effect of pollution on freshwater diatom communities was over a century ago (4) and afterwards—50, 60 years ago (5–7)—several authors proposed methodologies based on the taxonomic composition of diatom communities. In recent years, hundreds of studies have shown the usefulness of diatoms to monitor aquatic ecosystems (8). Nowadays, directives and laws require using this ecological indicator to routinely assess the ecological quality of rivers and lakes (e.g. in Europe with the Water Framework Directive (9) and in the US with the National Water-Quality Assessment Program (10)).

The diatom cell has the characteristic of being encased in two siliceous shells (valves), which are connected by girdle bands; together, the valves and girdle bands of a single cell comprise its ‘frustule’ (11). The current identification of diatom taxa is based on the morphology of the frustule. Standard procedures for diatom biomonitoring (e.g. for Europe: (12) are based on counting and determining several hundred of valves under the light microscope. This is time-consuming and requires a high level of taxonomic expertise. Moreover, distinguishing morphologically very similar taxa is difficult and can lead to misidentifications that compromise the accuracy of diatom index results for water quality assessment (13).

A solution that avoids these identification uncertainties and reduces analysis time is to replace microscopic identifications by molecular identifications based on DNA sequences. This is the concept of DNA-barcoding, a taxonomic method that uses a short genetic marker in an organism’s DNA to identify it as belonging to a particular species (14). This approach, first developed for animals, has recently been applied to diatoms (15, 16) and several DNA-markers were evaluated (18S, 28S, *cox*1, ITS, *rbc*L). Development of Next-Generation Sequencing (NGS) methods has opened a new area in the use of barcoding when applied to natural samples made of several taxa which is referred to as metabarcoding (17): NGS makes it possible to obtain a large quantity of data per sequencing run and by comparing each NGS sequence to the barcodes of a reference barcoding library, it enables the identification of the taxonomic composition of the natural community. The proof of this concept for diatoms has been shown first on mock communities—made of already barcoded strains—(18) and recently on natural communities from several temperate and tropical rivers (18, 19). These tests have shown that some barcodes yield better results than others. Cox1, displayed the most different molecular inventories from the expected inventories despite its high polymorphism distributed throughout the sequence: this is mostly due to the small number of reference barcodes built with Sanger sequencing. This small number is due to primer specificity that should be designed for each diatom genus. 18S (including v4 region) showed a good similarity between molecular and expected inventories mainly due to the highly variable v4 region and the high number of reference barcodes. The molecular inventories closest to expected inventories were obtained with rbcL because it showed a higher polymorphism than 18S with an equivalent number of reference barcodes. These tests highlighted also that an Achilles heel of metabarcoding was the reference barcoding library. It must be as complete as possible and requires a regular expert curation to maintain its quality (i.e. taxonomic homogeneity of assignations, sequence quality and traceability of data and metadata). Indeed the value of a curated database is to enable other workers to use it practically without having to sort out the same taxonomic name problems each time one is working with it. Several curated databases already exist, such as PR2 (20) or SILVA (21). They cover all microbes. But, a reference library dedicated to diatoms with a fine tuned taxonomy and curation at genus and species level was lacking.

In this article, we describe an open-access reference library, called R-Syst::diatom, and its curation procedures. For most of the freshwater taxa, phenotypic information is given (morphology, life-forms and ecological requirements).

This database was used in previous metabarcoding studies for river biomonitoring using diatoms (18, 22, 23). R-Syst::diatom is included in the French barcoding network R-Syst and gathers data for two barcodes (18S and *rbc*L). It is freely accessible through a website (http://www.rsyst.inra.fr/) and a supercomputing platform adapted for NGS analyses (Y.C. Laizet et al. 2014, in preparation) (https://galaxy-pgtp.pierroton.inra.fr/).

Data sources, metadata associated with the barcodes, data curation procedures, data storage and accessibility are presented in the methodology. Then results of a data curation exercise during an update of R-Syst::diatom and its contents are given and discussed.

## Materials and Methods

### Data sources

Two data sources are used to fill R-Syst::diatom: the barcoded strains of the Thonon Culture Collection (TCC) and the nucleotide database of NCBI.

#### Barcoded strains of the TCC

The UMR-CARRTEL is a research unit of the French National Institute for Agricultural Research (INRA) working on aquatic ecosystems. It has maintained the TCC since 1968, which is registered to the World Data Centre for Microorganisms (1030) and to the Global Registry Biorepository (http://grbio.org/institution/thonon-culture-collection-umr-carrtel-inra). A total of 858 monoclonal strains of freshwater microalgae are registered, among which 505 are diatoms. For each culture we keep in the laboratory of the UMR-CARRTEL, DNA extracts (25 ng/µl at −80°C) and raw material (living culture in growth chambers, frozen raw material in glycerin 50% at −80°C). Moreover, for diatoms, at least one permanent slide (Naphrax) of clean frustules as well as nitric acid treated material (in a vial) is kept. This material is accessible for subsequent studies. Two hundred eighteen diatom strains are maintained as live cultures in December 2015, the oldest was isolated in 1985 and the most recent in 2015. These strains are available on request through a website dedicated to the collection (http://www6.inra.fr/carrtel-collection_eng/). Each strain is sequenced for at least two barcodes: 18S and *rbc*L. Several research programs financed the isolations and sequencings (see Acknowledgements). All information about these strains, the sampling site location (georeferenced on a google map), the isolator, the barcode (including type of barcode, amplified region, primer used, protocols), the phenotypic data, the photos (all strains are photographed in light microscopy at ×40, ×100 in oil immersion and some of them in scanning election microscopy), the associated research programs (for sampling and sequencing) and its taxonomic affiliation are available on the R-syst website (http://www.rsyst.inra.fr/). The strains are identified using updated literature such as the entire collection of Diatoms of Europe, Iconographia Diatomologica, Bibliotheca Diatomologica and peer reviewed papers.

The TCC is regularly enriched with new isolated strains, which are sequenced for at least the two barcodes (18S, *rbc*L). Their entry in R-Syst::diatom is submitted to the curation process described in the section ‘Data curation’ here below.

#### Nucleotide database of NCBI

NCBI maintains a webserver that collects and provides molecular data and software. In particular, NCBI allows access to all public DNA sequence data via the GenBank database (24) (http://www.ncbi.nlm.nih.gov/genbank). We recovered all the nucleotide sequences of diatoms (freshwater and marine) available on GenBank main collection (CoreNucleotide) for the 18s (including V4 region) and *rbc*L whatever their length and their quality. We limited ourselves to these markers because they generally discriminate well between species and are therefore useful for species identification (25–28), they provide access to the largest taxonomic diversity and showed the best results for metabarcoding (18, 22). Sequences for other genes suggested as diatom barcodes – 28S and ITS rDNA and cox1—are not added to the database.

These sequences are retrieved regularly (every 6 months) using the following keywords on the Nucleotide Advanced Search Builder selecting ‘All fields’ in the drop-down menu: ‘(18s OR rbcL) and (diatom OR Bacillariophyta)’. In addition to these keywords, a publication interval in NCBI is indicated in the Advanced Search Builder selecting ‘Publication date’ in the drop-down menu: the oldest is corresponding to the last R-Syst::diatom update and the most recent to the current date. R-Syst::diatom is thus updated every 6 months. As well as the barcodes coming from the TCC, their entry in R-Syst::diatom is submitted to the curation process described in the next paragraphs.

In the particular case of a newly gathered sequences corresponding to uncultured and/or unidentified diatom in NCBI, those are not accepted in the database.

#### Phenotypic data

For most species, three kinds of phenotypic data are given: (i) morphological, (ii) life-form and (iii) ecological.

(i) Morphological data are gathering information about chloroplast and cell sizes. For the chloroplast, their shapes and number per cell are given for each taxon with the corresponding bibliographical references; most of the time, the publication of Cox (29) was used. When possible, photos of the strains were look at to get such information. Cell-dimensions (length, width, thickness), biovolume and size-class are given. Most of this information is derived from Rimet and Bouchez (30) which is a database gathering morphological and ecological information about freshwater diatoms. Omnidia (31) database which is gathering information about cell-biovolumes and sizes was also used. Original references where such information can be found are given.

(ii) Even if diatoms are basically unicellular algae, they exhibit an important diversity of life-forms, and many of them can form colonies. Taxa can even present several successive life-forms during their life-cycle (e.g. *Cymbella* can be unicellular and move freely at one time and attached to a peduncle and then immobile at another time). Different kinds of life-form information are documented in R-Syst::diatom (30), such as motility, kind of colony, type of attachment (pad, stalk, adnate, pedunculate).

(iii) Several kinds of ecological information are given. Nutrients, organic matter and moisture preferences of the species according to Van Dam *et al.* (32) are given. Habitat preferences (benthic, planktonic, epipsammic, epipelic) are given mostly according to Round *et al.* (11). The ecological guilds to which species belong (high-profile, low-profile, motile, euplanktonic) are given according to Rimet and Bouchez (30). Finally, the pollution sensitivity values and ecological weights of several diatom indices are given, such as the TDI (Trophic Diatom Index) (33), the TDI-Sweden, (34), the IPS (Pollution Sensitivity Index) (35) or the Phylogenetic-IPS (36).

### Data curation

The identifications and sequencing of diatoms included in R-Syst::diatom were carried out by different people and may not be equally reliable. There are three important drawbacks to take into account when gathering new sequences in R-Syst::diatom:

- First, in NCBI, data were deposited by different authors at different times: the first data were deposited in 1998. From this date to the present, taxonomy has evolved.

- Second, the identifications and taxonomic skills of the different authors who deposited their data in NCBI can be heterogeneous. The same problem is also visible for TCC.

- Third, the length or the quality of the sequences cannot be adapted for correct taxonomic affiliation.

These three drawbacks underline the necessity to curate the taxonomic names of the strains and their corresponding sequences in order to have homogeneous taxonomic names in R-Syst::diatom. As diatom taxonomy is under active development, the aim is to achieve for similar sequences a similarity in their taxonomic names, and to ensure that these names are as taxonomically correct as possible according to the most recent taxonomy literature. However, as diatom taxonomy is under active development, there will be cases where only a consensus for practical use can be made and solutions regarding the correct name will have to await further scientific studies. In any case, if the original taxonomic name given by the authors of the sequence is changed during the curation procedure, the traceability of the original name is kept in the database and is visible on R-Syst web portal.

This data curation is carried out in three steps (the first two steps are mandatory):

- The first step is pre-curation. The objective of this first step is to check if each newly retrieved sequence from NCBI or the TCC has a comparable taxonomic name to similar sequences formerly deposited in NCBI, and to check if the quality and length of these sequences is correct. For this purpose, the newly retrieved sequences are compared to the entire NCBI database using Blast.
- The second step is detailed curation. The objective of this second step is to compare the new sequences meeting the criteria of the first step with the sequences already included in R-Syst::diatom, based on a local alignment methodology, called ‘Declic algorithm’ (for detail see ‘Second curation step’ section of this ‘Data curation’ part). If these sequences have taxonomic names similar to comparable sequences then their taxonomic names and the sequences are kept for the third curation step. If the taxonomic names from comparable sequences are different, then the taxonomic names are checked through a taxonomic curation procedure.
- The third step is an optional curation (for reasons see ‘Third curation step’ of the ‘Data curation’ part). The objective is to compare the new sequences meeting the criteria of the second step with those already included in R-Syst::diatom database, based on a global alignment and phylogenetic analyses. If the taxonomic names from comparable sequences are different, then the taxonomic names are checked through a taxonomic curation procedure. The second and third curation step are based on different algorithms which both have different advantages and so are complementary. The Declic analysis is based on local alignment and is run on all sequences whatever their length. The phylogenetic analysis is based on global alignment and is run on a sub-set of sequences which have a long common covering (1000 bp at least). Declic has the advantage to compare all sequences whereas the phylogenetic analysis has the advantage to compare with better precision a sub-set of sequences.

**Figure 1.**
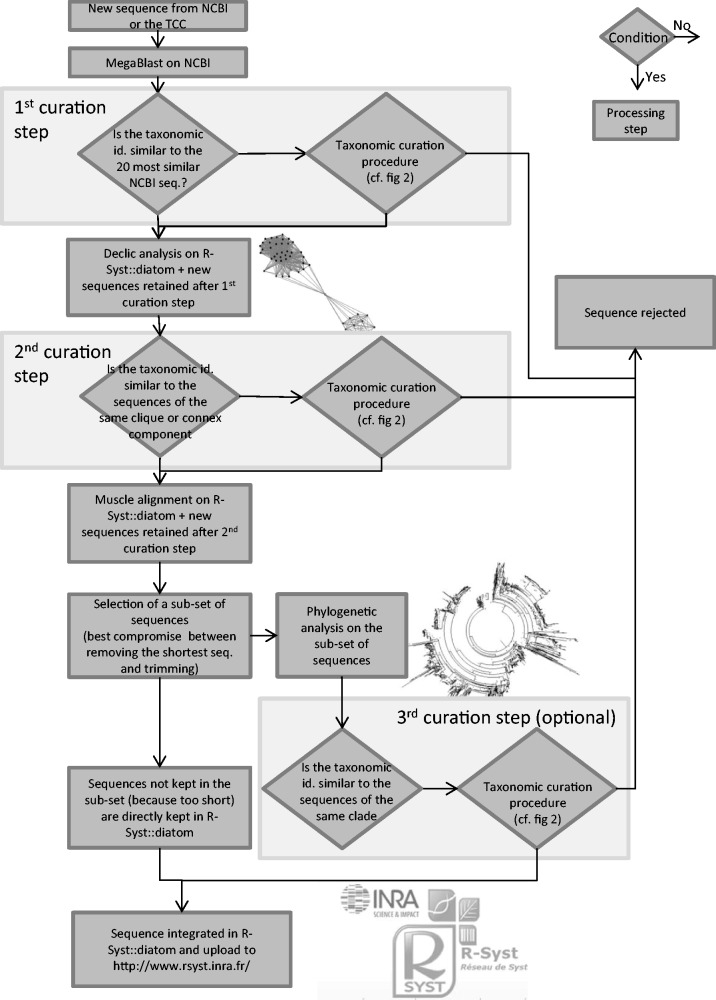
General flowchart of the curation and integration of new sequences in the R-Syst::diatom. Taxonomic curation procedure is detailed in a flowchart (Figure 2). Diamonds are conditions, the arrow from the bottom point of the diamond corresponds to ‘Yes’, the arrow from the right point of the diamond corresponds to ‘No’. Rectangles are processing steps.

**Figure 2.**
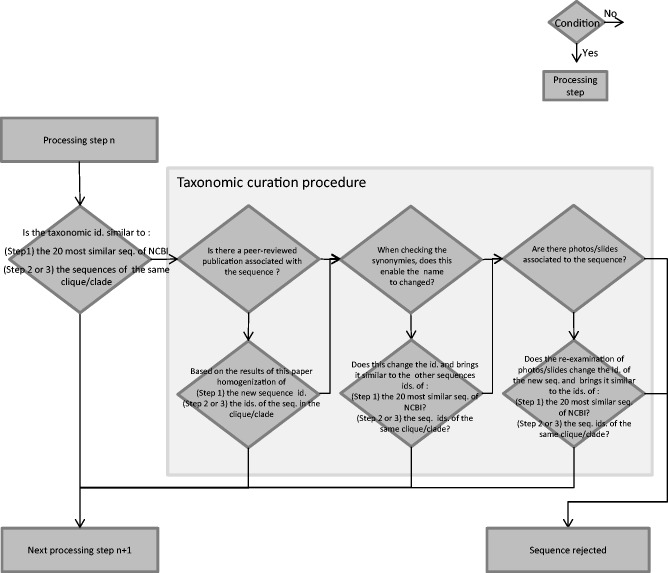
Flowchart of the taxonomic curation procedure. Diamonds are conditions, the arrow from the bottom point of the diamond corresponds to ‘Yes’, the arrow from the right point of the diamond corresponds to ‘No’. Rectangles are processing steps.

Figure 1 gives an overview of the general workflow of the curation procedures. Figure 2 gives details on the taxonomic curation procedure which is used several times in the general workflow of Figure 1.

#### First curation step: pre-curation using NCBI and Blastn of each sequence

For each sequence (whatever its quality or length), newly gathered from NCBI or coming from new strains of the TCC, a Blastn is run on the entire NCBI database. The 20 sequences showing the best pairwise identity matching to this new sequence are consulted.

If the taxonomic affiliation of the new sequence is close to those of the 20 other sequences then the taxonomic affiliation is kept and this new sequence is kept for the second curation step. Taxonomic affiliation designated here is not necessarily the species level: it can be the genus or family level in the case of newly isolated genera or families never isolated before. For instance, if the newly retrieved sequence is identified in an already well sequenced genus (e.g. *Pseudo-nitzschia* or *Fistulifera*), the 20 closest sequences should belong to the same genus, and even to the same species if the species has been already sequenced formerly (e.g. *Nitzschia palea, Cyclotella meneghiniana*). On the other hand, if the new sequence is named with a genus which has never been sequenced before, it is expected that the 20 closest sequences belong to the same family or order (e.g. sequences of *Didymosphaenia* had to be close to sequences belonging to Cymbellales).

If there is discordance between the taxonomic affiliation of this new sequence and those of the 20 other sequences, then the taxonomic curation procedure is applied (Figure 2):

- First, we check if a peer-reviewed publication is associated with this new sequence. In this case this new sequence and its taxonomic name are kept for the second curation step. If it is not the case, then point (ii) of the taxonomic curation procedure is considered.
- If no peer-reviewed publication is available, taxonomic synonymies are checked using Algaebase website (http://www.algaebase.org/) (37), the catalogue of diatom names of E. Fourtanier and P. Kociolek (http://researcharchive.calacademy.org/research/diatoms/names/index.asp) or Omnidia software (31). If this enables the homogenization of its taxonomic name, then the new sequence and its new taxonomic name are kept for the second curation step. If it is not the case, then point (iii) of the taxonomic curation procedure is considered.
- If no peer-reviewed publication exists, and if the taxonomic synonymies check was not successful, we check if some photos or slides associated to the sequence are available (e.g. in TCC or Algaterra databases (38) or websites of culture collections such as http://www.ccap.ac.uk/). If the re-examination of this material (photos/slides) shows that the strain was wrongly identified then a correct taxonomic name is given. If this new taxonomic name is similar to those of the 20 most similar NCBI sequences, this sequence is kept for the second curation step. If it is not the case, no photos/slides are available or the new taxonomic name still differs from those of the 20 most similar sequences, then this new sequence is not accepted in the database.

After gathering all the new sequences from the first curation step, additional curation steps are done by comparing them to the sequences already in the R-Syst::diatom database. Two different and complementary tools are used. The first tool is Declic analyses (second curation step). This analysis is based on local alignments which are useful when sequences of dissimilar sizes have to be compared. If this is the case of the data usually gathered: depending on the authors, only parts of 18s/rbcl are sequenced. The second tool is phylogenetic trees (third optional curation step) based on global alignments. Global alignments are more useful when sequences of similar sizes are compared and are carried out on a sub-set of sequences of homogeneous size and similar regions.

#### Second curation step: use of Declic on the entire database

**Figure 3.**
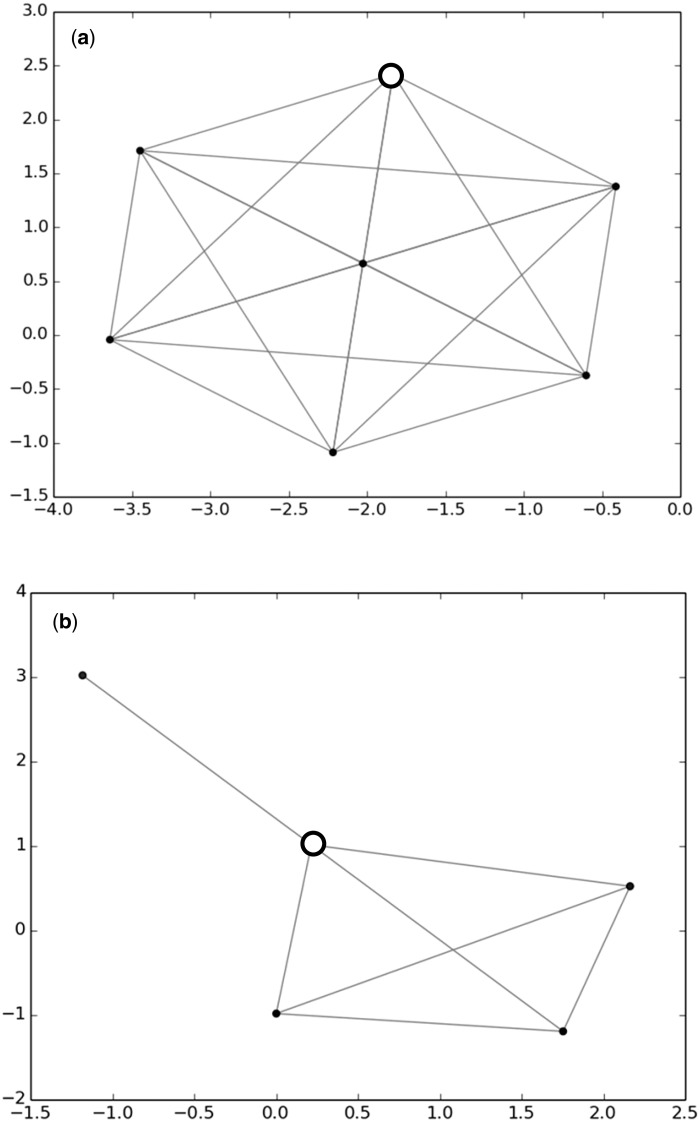
Use of Declic analyses to curate the database: case of taxonomically heterogeneous clique (a) and connex component (b). (a) *Gomphonema bourbonense* clique (18s, gap 8) with one *G. angustum* sequence (TCC460)—white circle and (b) *Encyonema* spp. connex component (18s, gap 8) with one *Craticula cuspidata* sequence (KM084917)—white circle. TCC460 strain identification was changed into *G. bourbonense* after checking photos. KM084917 was rejected since there is an obvious mistake of identification.

**Figure 4.**
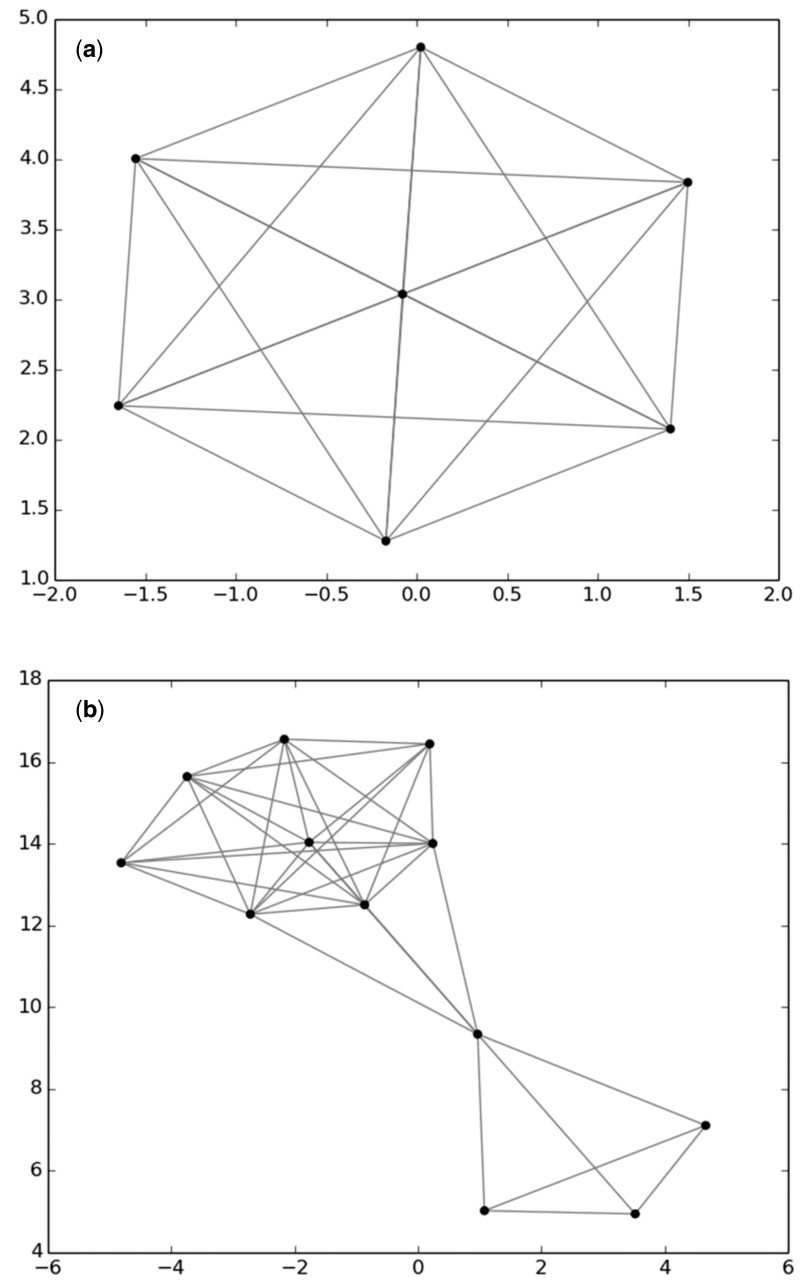
Use of Declic analyses to curate the database: case of taxonomically homogeneous clique (a) and connex component (b). (a) *Pseudo-nitzschia multistriata* (b) *Pseudo-nizschia delicatissima*. No changes were made in these cases.

In the second curation step, the newly retrieved sequences and the sequences of the R-Syst::diatom database are compared two by two by mean of Declic (for Delimitation of species with cliques) software written in python (19) which enable (i) to define OTU (Operational Taxonomic Units) through an unsupervised clustering algorithm and (ii) to represent these OTU in a two dimensional space. This software can be run with an R-package (39) or under a galaxy platform (https://galaxy-pgtp.pierroton.inra.fr/) within the Virtual BiodiversityL@b folder (Y.C. Laizet et al. 2014, in preparation). Briefly, Declic analysis is run after computing pairwise local alignment scores (40) which are then transformed into distances. We then have a full pairwise distance matrix. Pairwise distances can be visualized by running Multi Dimensional Scaling (MDS) on the distance matrix. Second, a graph is attached to the matrix, where the nodes are the sequences, and there is an edge between two nodes if the distance between the two sequences is lower than a given threshold (one graph per threshold). The threshold is selected by the user and a graph can be built for any threshold value. The graph is projected onto the plane using the Fruchterman–Reingold layout (41). If edit distances were evolutionary distances, and if a threshold exists for separating taxa, then a taxon would be a clique (i.e. in this case a subset of sequences which are all connected with each other by an edge, see Figure 3a). As we have edits distances from best local alignment, we built the connex components (i.e. in this case a subset of sequences which are connected by at least one edge, see Figure 4b) of such a graph, expecting they are close to cliques, and related to taxa. Such a threshold may play the role of a barcoding gap, although some sequences within a connex component can be at a distance larger than the gap. Colors are given to sequences belonging to the same taxon. The taxonomic levels which are selected for data curation are genus level or species level. A threshold of 1% is usually considered to separate diatom species (15, 26), nevertheless generally a threshold below 1% is selected during this curation procedure because different species (e.g. *Fragilaria capucina, perminuta, tenuistriata*) and even genera (e.g. *Surirella, Campylodiscus*) often merge in the same group with 1%.

It is expected that a new sequence has a homogeneous taxonomic name with the other sequences of the clique it belongs to. If it is the case, the sequences of this clique and their taxonomic names are kept for the third curation step.

If the new sequence and the other sequences have heterogeneous taxonomic names inside the same clique, then the taxonomic curation procedure is applied (Figure 2) in a similar way as in the first curation step:

- We check if a peer-reviewed publication is associated with the new sequence. If it is the case, based on the results of the publication, the taxonomic names of the sequences are homogenized in the clique and the sequences are kept for the third curation step. If no peer-reviewed publication is available, then point (ii) is considered.
- In the case that there is no publication available for the new sequence, the synonymies of the taxonomic names of the sequences inside this clique are checked (using i.e. Algaebase, Catalogue of diatom names, Omnidia). If this enables us to homogenize the taxonomic names, the sequences and their new taxonomic names are kept for the third curation step. If this is not the case, point (iii) is considered.
- If no publication is associated to this new sequence and if the synonymies check did not enable us to homogenize taxonomic names, we check if photos/slides associated to it (collections as TCC, Algaterra, Bold) are available. If the re-examination of the photos/slides enable us to change the taxonomic names and make it similar to those of the other sequences in the clique, the sequence and its new taxonomic name is kept for the third curation step. If the taxonomic name is still different after checking photos/slide, the sequence is rejected.

Connex components which were not cliques are also checked. Sequences belonging to the same connex component should show homogeneous taxonomic names. If not, the same procedure (described here above) for cliques was adopted (check of literature, synonymies, photos …).

#### *Third* *curation step (optional): phylogenetic analyses*

As with the Declic analyses, phylogenetic analyses are carried out for each marker (18S and *rbc*L) on all sequences (new sequences and R-Syst::diatom). A general alignment is carried out on all the sequences with Muscle in Seaview (42). The best compromise between removing the shortest sequences and trimming the alignment is found in order to keep an alignment long enough to get phylogenetic analyses robust enough. Usually, for 18S and *rbc*L the alignment is carried out on 1000 bp at least and so shorter sequences or sequences which have <1000 bp in common with the other sequences are not taken into account in this curation step. From this general alignment, all sequences are trimmed at the same length and a neighbor joining tree is run with Seaview (42) or Mega5 (43). The same verifications as those carried out in the second curation step are done: if taxonomic names in a given clade are heterogeneous, the taxonomic curation procedure is applied (Figure 2).

These phylogenetic analyses are done to confirm the curation completed with Declic analyses. Nevertheless, phylogenetic analyses are carried out on a sub-set of the database only, since short sequences are not integrated in this analysis. The shortest sequences, which were not integrated in the phylogenetic analyses, are only curated with the Declic analyses and if they meet all the criteria of taxonomic homogeneity in the second curation step they are directly integrated in R-Syst::diatom.

### Data storage and open access

All the curated data are stored in a PostgresSQL database built in the frame of the R-Syst network. R-Syst is a collaborative network supported by INRA for studies in systematics. It comprises several tens of research teams including technicians, researchers and engineers in the fields of molecular biology, genetics and bioinformatics who are involved in the molecular and morphological characterization of organisms. Among those, micro-algae are represented and a dedicated web interface is available from the R-Syst web portal (http://www.rsyst.inra.fr/en) to browse the stored data of the diatom barcoding database.

On this website, the algae section of the database gathers information about diatom strains (but also about Chlorophyta and Cyanophyta strains of the TCC) which were characterized for three kinds of criteria: taxonomic, phenotypic and genetic.

For each strain, the following information is given when available: (i) sampling site (name and location on Google map), (ii) type of habitat, (iii) strain code given by the laboratory, (iv) name of the project which funded the field sampling, sequencing, (v) laboratory responsible for field sampling, (vi) DNA extraction, (vii) PCR, sequencing and (viii) the dates of the different steps. A species name is given to each strain, except in a few cases where only genus level is given. Moreover the taxonomic affiliation is given until the regnum (11, 37, 44). For molecular criteria, the database gives the type of marker (18S or *rbc*L), the primers used for sequencing and PCR. Protocols for DNA extraction and PCR are also given. The laboratory responsible of the sequence is given. For phenotypic information, photos (living material and empty frustules) of the TCC strains are given.

## Results and Discussion

### Examples of curation

Results of the curation process of R-Syst::diatom in January 2015 are given in Table 1 and supplementary files give the list of sequences whose taxonomical name was changed after curation (Supplement data 1) and those which were not integrated in R-Syst::diatom (Supplement data 2). This curation was carried out on new sequences downloaded from NCBI and coming from the TCC between 29 July 2014 and 16 January 2015.

**Table 1.** Results of the curation procedure of 16 January 2015

Curation steps		18s	rbcl
	New sequences (TCC—NCBI)	428	194
	R-Syst::diatom (former version)	1911	1624
First curation step	Sequences having a different identification from the 20 most similar NCBI sequences	207	4
	Sequence published in peer review paper	45	1
	Check of photos -> modification of the determinations	0	0
	homogenization of taxonomy/synonymies	0	0
	Sequences rejected	162	3
	Sequences kept	45	1
	New sequences after first curation step	266	191
Second curation step	Sequences having a different identification from the sequences of the same clique	16	32
	Changes according to peer review papers	3	3
	Check of photos -> modification of the determinations	6	6
	Homogenization of taxonomy/synonymies	5	21
	Sequences rejected	2	2
	Sequences kept	14	30
	New sequences after second curation step	264	189
Third curation step	Sequences having a different identification from the sequences of the same clade	9	10
	Changes according to peer review papers	3	2
	Check of photos -> modification of the determinations	0	5
	Homogenization of taxonomy/synonymies	1	3
	Sequences rejected	5	0
	Sequences kept	4	10
	New sequences after third curation step	260	189
	R-Syst::diatom (new version)	2171	1813

Sequences were imported from NCBI and the TCC between 29 July 2014 and 16 January 2015. Values in the table give the number of sequences.

*First curation step:* In the curation process carried out in January 2015 (Figure 1), several sets of sequences deposited in NCBI were not kept because determinations were clearly erroneous or insufficient. This was especially the case for 18S where 162 sequences were rejected because of an insufficient taxonomic identification (class level identifications).

*Second curation step: *Taxonomic names of several sequences were modified after Declic both for 18S and *rbc*L. Modifications were for instance performed on a clique where all 18S sequences belonged to *Gomphonema bourbonense* except one *Gomphonema* *angustum* (Figure 3a). All these sequences came from the TCC, which meant that the identities of the strains could be checked from our photos. After checking the photos of *G. angustum* (strain number: TCC460), we realized that the determination was erroneous, and the name was changed to *G. bourbonense.* A second example is a connex component, which gathered five sequences of *Encyonema*, except one which belonged to *Craticula.* These two genera are very different morphologically and phylogenetically and cannot be in the same connex component. Even if though the sequence was recently published in a peer reviewed paper it was rejected. As an example of a no-change, we show the connex component (Figure 4a) and the clique (Figure 4b) for two species of *Pseudo-nitzschia*, which names were taxonomically homogeneous.

### Content of the database

#### Number and length of sequences

The number of sequences available for 18S and *rbc*L is given in Table 2. Sequences from the TCC represent 21% of the total number of sequences for 18S, and 19% for the *rbc*L. Only 16.7% of the 18S sequences of the TCC have been deposited on NCBI and have an accession number. Similarly, 21.7% of the *rbc*L sequences of the TCC were deposited on NCBI. These sequences were deposited in the framework of several peer-reviewed publications, as phylogenetic studies (45, 46), metabarcoding analyses using NGS (18, 22), diversity studies (47), ecotoxicological studies (48). The objective is to submit all accepted sequences from the TCC on NCBI.

**Table 2.** Number of sequences in the R-Syst::diatom database coming from NCBI and the TCC (December 2015)

Marker	18S	18S-V4^a^	rbcL
No. of sequences from the TCC	468	—	373
No. of sequences from NCBI	1759	85	1613

^a^ le 18S-V4 is a region of 18S. It is counted is a separated column because these sequences are limited to this particular region.

**Figure 5.**
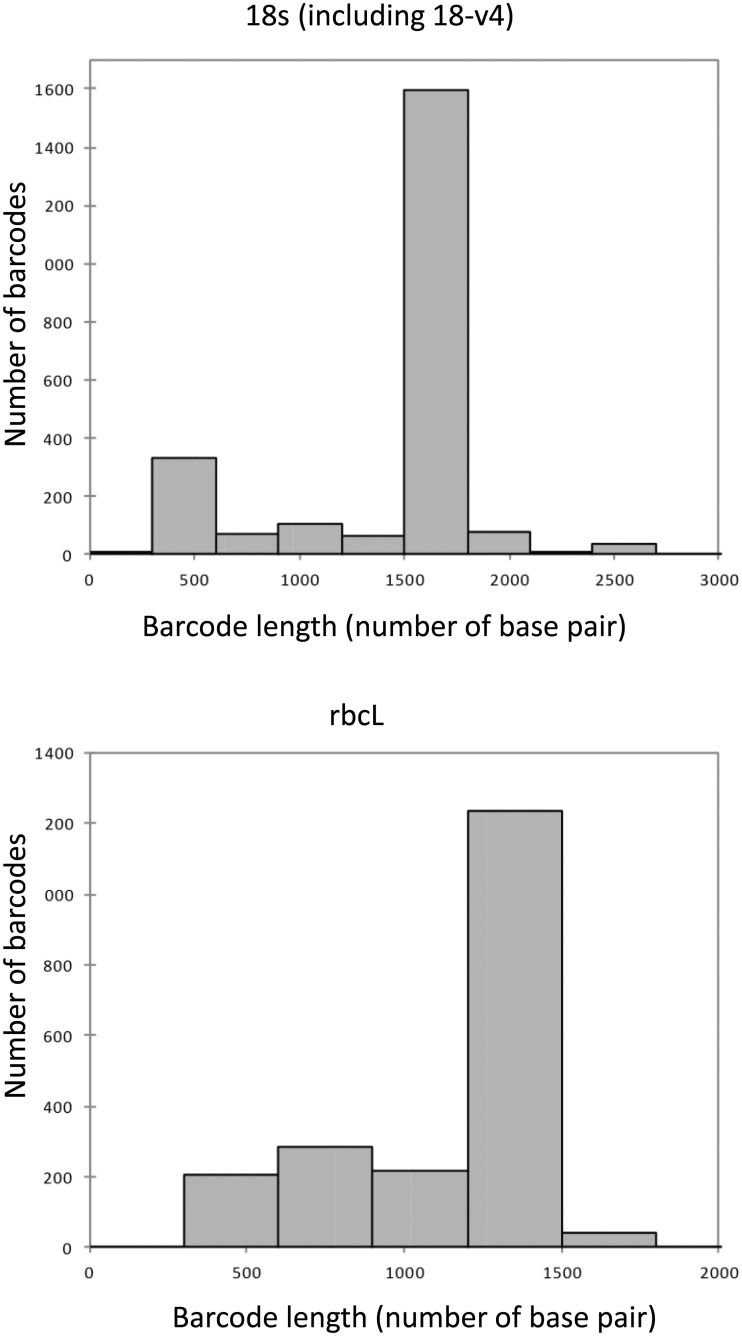
Amount and length of the barcodes present in R-Syst::diatom (update of September 2015).

The lengths of the barcodes in R-Syst::diatom is given in Figure 5. For 18S, a large majority of the sequences have lengths of 1600–1800 bp. Several of them are however much shorter (400–500 bp) and correspond to the 18-v4 region proposed as barcode by Zimmermann *et al*. (28) for species identification. For *rbc*L, a large majority of the sequences have lengths of 1200–1600 bp. A few of them are much shorter and correspond to sequences deposited in the framework of barcoding studies studying the efficiency of shorter fragments inside the *rbc*L for species identification (e.g. *rbc*L 3P see Refs. 27 and 50), or were mostly submitted in the framework of the phylogenetic studies of Bruder and Medlin (50, 51).

#### Taxonomic coverage of diatom taxa

**Figure 6.**
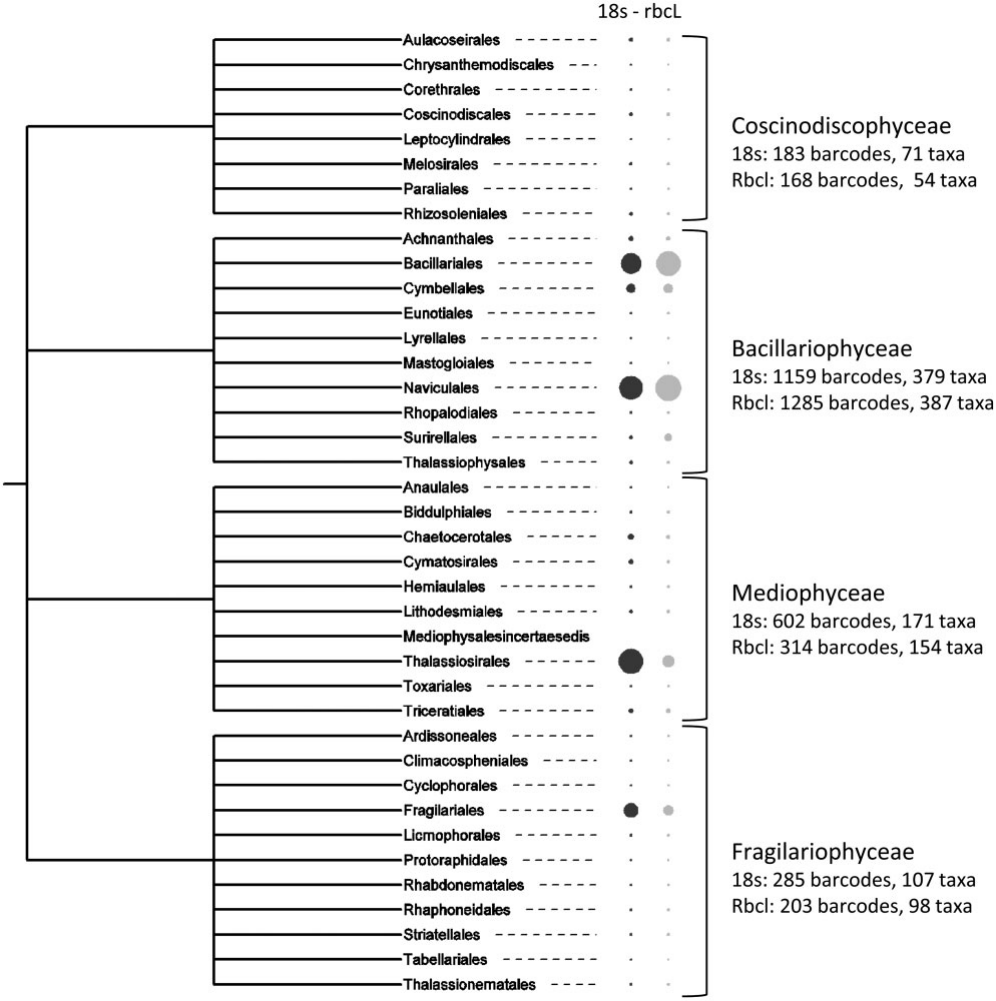
Number of barcodes (18S and rbcL) in the R-Syst::diatom for the different diatom classes and orders (update of September 2015). Large circles correspond to high number of barcodes. Tree created with itol.embl.de.

To show the distribution of barcodes in the diatom taxonomy, we followed the classification given by Medlin (52) with the three classes Mediophyceae, Coscinodiscophyceae, Bacillariophyceae, and we kept the Fragilariophyceae as described in Round *et al*. (11), which were recently shown to be non-monophyletic (except Bacillariophyceae) but were grades which evolved from radials (Coscinodiscophyceae) to polar (Mediophyceae) to araphids (Fragilariophyceae) and then to raphids (Bacillariophyceae) (53). Figure 6 gives an overview of the number of barcodes and taxa for each class in the R-Syst::diatom database. The Bacillariophyceae is the most barcoded class with the Bacillariales and the Naviculales orders. In all diatom classes the number of barcodes is higher for 18S than for *rbc*L: this can be explained by the longer legacy of 18S in diatom phylogeny (54 and 55), than *rbc*L which started to be used in diatom barcoding and phylogeny more recently (25).

**Figure 7.**
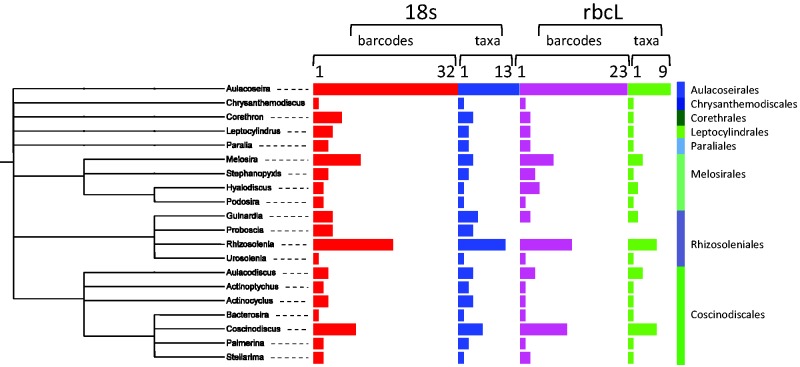
Number of barcodes (18S and rbcL) and taxa in R-Syst::diatom for Coscinodiscophyceae orders (update of September 2015). Red and purple horizontal bars give the number of barcodes, respectively, for 18S and rbcL. Blue and green horizontal bars give the number of taxa, respectively, for 18S and rbcL. Tree created with itol.embl.de based on diatom taxonomy.

In the Coscinodiscophyceae (Figure 7), the most sequenced genus is *Aulacoseira* (respectively, 31 and 23 for 18S and *rbc*L sequences). *Aulacoseira* strains are mostly sequenced by Shcherbakova (56), Edgar and Theriot (57) and Medlin and Kaczmarska (58).

**Figure 8.**
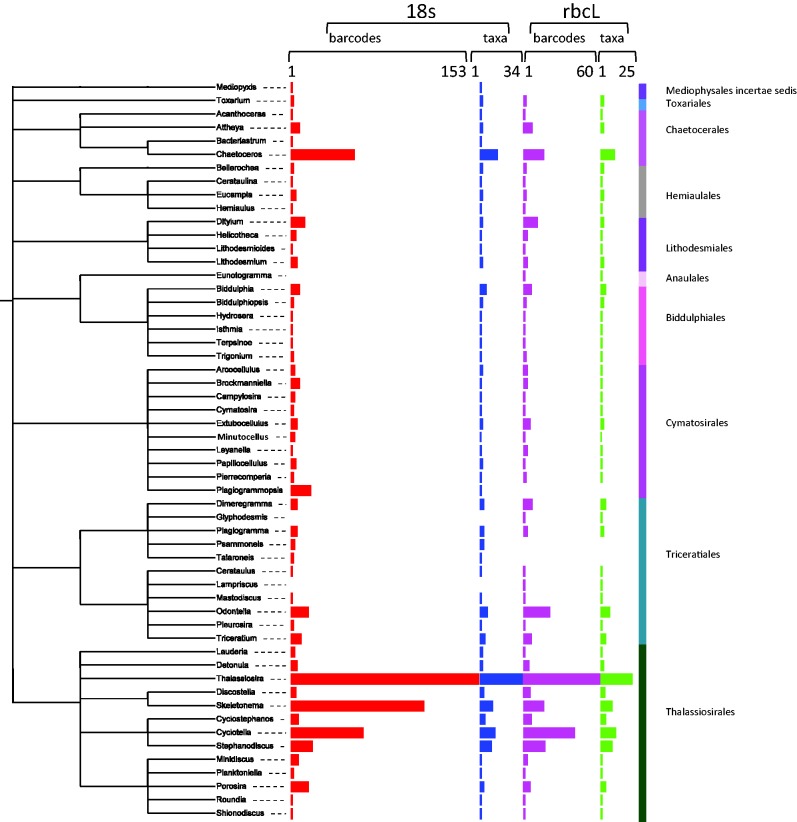
Number of barcodes (18S and rbcL) and taxa in R-Syst::diatom for Mediophyceae orders (update of September 2015). Red and purple horizontal bars give the number of barcodes, respectively, for 18S and rbcL. Blue and green horizontal bars give the number of taxa, respectively, for 18S and rbcL. Tree created with itol.embl.de based on diatom taxonomy.

In the Mediophyceae (Figure 8), the most sequenced genera are the *Thalassiosira* (respectively, 151 and 61 for 18S and *rbc*L sequences), *Skeletonema* (respectively, 107 and 16 for 18S and *rbc*L sequences) and the *Cyclotella* (respectively, 58 and 41 for 18S and *rbc*L sequences). *Thalassiosira* are intensively studied by Alverson et al. (59), Luddington et al. (60), Whittaker et al. (61) among others. The *Skeletonema* genus is also often sequenced (62–64).

**Figure 9.**
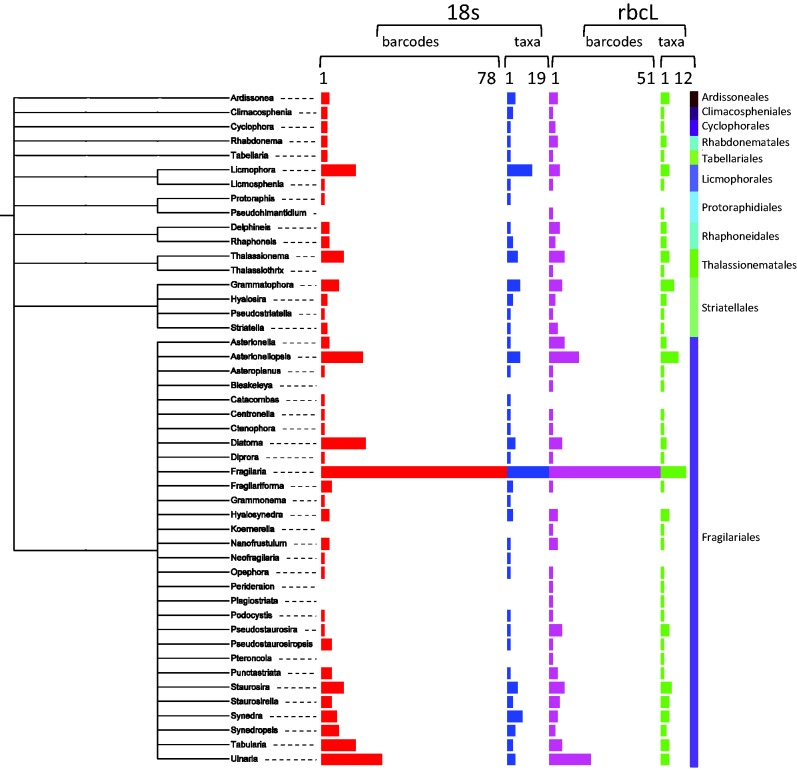
Number of barcodes (18S and rbcL) and taxa in R-Syst::diatom for Fragilariophyceae orders (update of September 2015). Red and purple horizontal bars give the number of barcodes, respectively, for 18S and rbcL. Blue and green horizontal bars give the number of taxa, respectively, for 18S and rbcL. Tree created with itol.embl.de based on diatom taxonomy.

In the Fragilariophyceae (Figure 9), the most sequenced genus is *Fragilaria* (respectively, 77 and 46 for 18S and *rbc*L sequences), the other genera are much less represented in the database: *Asterionellopsis* (respectively, 17 and 22 for 18S and *rbc*L sequences) and *Diatoma* (respectively, 18 and 5 for 18S and *rbc*L sequences). The majority of the *Fragilaria* sequences are coming from the TCC collection and are not yet published (all photos and related data are available of R-Syst website).

**Figure 10.**
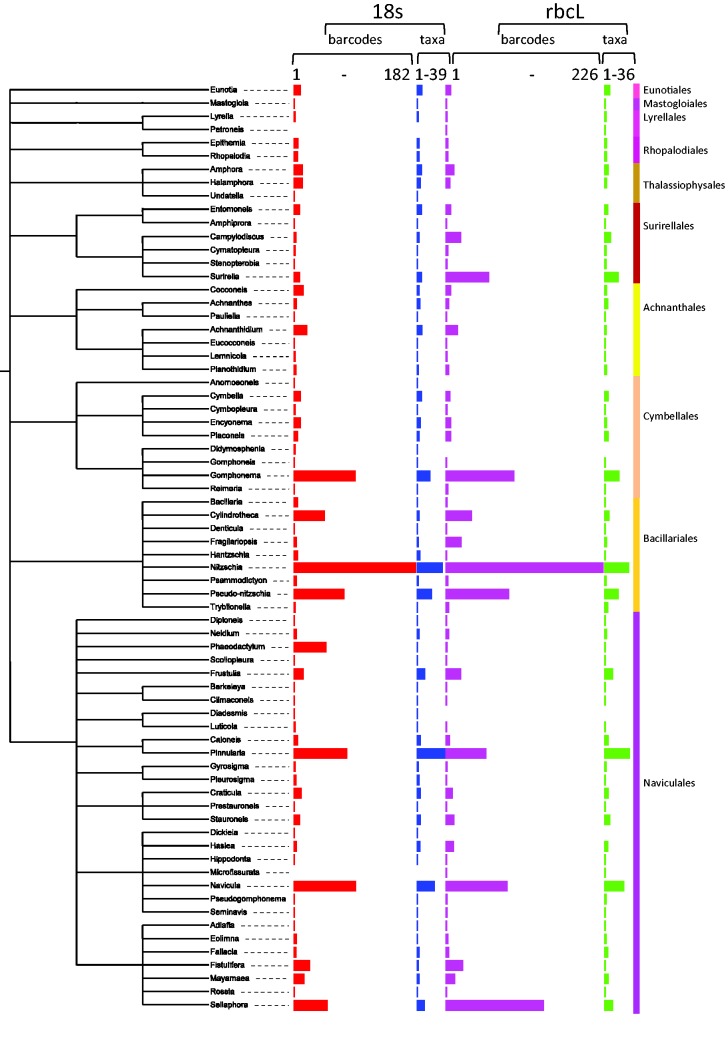
Number of barcodes (18S and rbcL) and taxa in R-Syst::diatom for Bacillariophyceae orders (update of September 2015). Red and purple horizontal bars give the number of barcodes, respectively, for 18S and rbcL. Blue and green horizontal bars give the number of taxa, respectively, for 18S and rbcL. Tree created with itol.embl.de based on diatom taxonomy.

In the Bacillariophyceae (Figure 10), the most sequenced genera are *Nitzschia, Navicula, Gomphonema, Pinnularia, Pseudo-nitzschia, Sellaphora.* In particular, *Nitzschia palea*, an indicator of polluted freshwaters, is the most sequenced species (respectively, 66 and 88 for 18S and *rbc*L sequences), because it is intensively studied (47, 65, 66). Similarly, *Gomphonema parvulum*, which is also an important freshwater quality indicator species, is intensively barcoded (respectively, 51 and 54 for 18S and *rbc*L sequences) by Kermarrec et al. (27) and Abarca et al. (67). The *Sellaphora* genus is also a very well-studied genus, in particular the *Sellaphora pupula* species complex since it was studied as well for its reproduction mode than its phylogeny (68–70) and has been a model for barcode tests (25). *Pseudo-nitzschia* genus, a potential harmful diatom genus which can bloom in marine waters is often investigated. Several studies, based on genetic data, show a cryptic diversity inside several species (49, 71, 72). Several species belonging to the *Navicula* have been sequenced in several papers (50, 51, 73–75). *Pinnularia* genus is mostly studied by Souffreau et al. (76).

#### Phenotypic data and their usefulness for ecological assessment

Greater than 93% of the barcodes present in R-Syst::diatom have associated a phenotypic information, only a few of them (7%) have none. These 7% correspond to marine taxa which were recently described. Since R-Syst::diatom objective is mostly devoted for freshwater ecological assessment we did not looked actively for information about such kind of taxa; nevertheless marine diatom taxa are integrated in R-Syst::diatom since rivers and lakes can present marine taxa because of industrial wastes or natural situations (77).

Information about life-form (motility, kind of colony, kind of attachment), habitat preferences (planktonic, benthic, epipelic, epipsammic…), ecological guild (motile, low-profile, high-profile, euplanktonic) is given for the species identification of the barcodes. Similarly, in 93% of the cases, chloroplast shape, number and bibliographical references are given. Cell-dimensions and biovolumes are given for 67% of the barcodes. The diatom indices values are given for 72% of the barcodes for the IPS and 49% for the TDI and 40% for the TDI-Sweden, because a large part of the barcodes are coming from marine habitats which are not covered by these diatom indices devoted to freshwater ecosystems. Similarly, only 38 to 40% of the barcodes have information for the ecological classes of nutrients, organic and moisture requirement of Van Dam et al. (32).

Several papers have shown the usefulness of diatom indices (8, 78, 79), ecological guilds and life-forms (80, 81) for lake and river assessment using classical microscopic data. Unpublished ongoing work (V Vasselon et al., personal communications) show that using similar metrics (diatom indices, life-forms) with metabarcoding data can also give a robust and accurate assessment of ecological quality of freshwater ecosystems. This is why it appears necessary to integrate this kind of phenotypic and ecological information to move forward to an environmental assessment using metabarcoding technology.

## Conclusion

In their article, Zimmermann et al. (82) highlight the importance of the quality of reference barcoding libraries. In particular, the traceability and availability of the metadata (sampling site, isolation protocols, pherograms, vouchers, slides, DNA, photos, etc …) and the physical deposit of vouchers (culture, raw material, slides, DNA, etc…) associated with the barcode are necessary for accurate biodiversity studies. Such requirements were recently integrated into a pre-standard protocol by the European Committee for Standardization (83). Indeed, several European laboratories (in France, Germany, Hungary, United-Kingdom, Spain, Czech Republic, Belgium …) working on diatom barcoding for biomonitoring have agreed on a minimum set of metadata that must accompanied a normative barcode for a particular diatom taxon. Diatom barcodes stored in databases such as R-Syst, Algaterra of Bold fulfill these gold requirements.

Moreover, Zimmermann et al. (82) say that barcodes ‘lacking voucher specimens … are of no future use and valuable information is lost to science’. Our position is slightly different: historical data stored in NCBI may not meet the criteria now being established in the pre-standard protocol but are nevertheless precious and are not all ‘lost to science’. Indeed, getting a clonal culture from a natural sample and then growing it until getting enough biomass to extract its DNA, to mount it for permanent slide, to treat it for scanning electron microscopy is a long and risky process (many cultures are lost before getting DNA or permanent slides). Not considering barcodes that are not backed by voucher specimens will in some cases waste valuable data for barcode reference libraries: some such barcodes have been published in peer reviewed journals and are accompanied by photographs and other metadata. These, if carefully evaluated as described in this article, before sequences are integrated into the database, can be valuable, filling gaps in the taxon coverage.

Routine molecular identification of diatom taxa in natural communities to a genus level (instead to a species level) with R-Syst::diatom is not a problem (18, 22, 84) because taxon coverage of the reference library at a genus level in freshwaters is almost complete. But, for molecular identification at a species level, even using all possible barcodes and after curating them (as shown in this article) before their integration in R-Syst::diatom, the library still suffers from an underrepresentation of some taxa living in particular habitats. In particular, pristine freshwater habitats and tropical rivers should be sampled more frequently to get cultures and to barcode them. This is a challenge of the next few decades.

Another difficulty met in the database is that several sister species—the definition and delimitation of which are classically based on morphological criteria—display an overlap when considering their barcodes proximity. The result is that such species could be paraphyletic . This is currently the case for species in the *Fragilaria capucina* group (*capucina, perminuta, nanoides, gracilis* …) when considering their 18S or rbcL barcodes. This type of problem also occurs, but less often, for generic levels (e.g. *Surirella, Campylodiscus*). Our curation procedure is not sufficient in these cases and an integrative study based on genetic data (using multiple markers), morphological data (scanning electron microscopy, light microscopy) and also ecological data must be undertaken to adjust taxonomy. A good example of such integrative studies is shown in the sister species *Nitzschia soratensis/inconspicua* (85, 86). More studies of this kind are required for the development of the database, in order to present well-defined and monophyletic taxa so that the molecular identifications of sequences from NGS can be done easily.

Finally, barcode databases have until now linked sequences only to a taxonomic identification, together with sufficient metadata to ensure data traceability and sequence origin (e.g. Barcoding of Life Data Systems (BOLD), http://www.boldsystems.org/). However, this information is not enough to move forward to a routine biomonitoring with metabarcoding. Ecological functions or requirements (e.g. diatom indices values in our case) of taxa are unfortunately not provided in the existing barcoding databases. Providing such information in R-Syst::diatom should enable a routine use of metabarcoding for next-generation biomonitoring (87).

## Supplementary data

Supplementary data are available at *Database* Online.

Supplementary Data
