# Supplementary material for: R-Syst::diatom: an open-access and curated barcode database for diatoms and freshwater monitoring
Source: Database (Oxford). 2016 Mar 17;2016:baw016. doi: 10.1093/database/baw016 (PMC4795936; doi:10.1093/database/baw016)
Supplement: Supplementary Data [file supp_2016_baw016_index.html]

Supplementary Data 

# R-Syst::diatom: an open-access and curated barcode database for diatoms and freshwater monitoring

## Supplementary Data

files

- Supplementary Data - xlsx file
- Supplementary Data - xlsx file
